# Construction site workers’ malaria knowledge and treatment-seeking pattern in a highly endemic urban area of India

**DOI:** 10.1186/s12936-016-1229-2

**Published:** 2016-03-16

**Authors:** Siddharudha Shivalli, Sudarshan Pai, Kibballi Madhukeshwar Akshaya, Neevan D’Souza

**Affiliations:** Department of Community Medicine, Yenepoya Medical College, Yenepoya University, Mangalore, Karnataka 575018 India; Department of Community Medicine, Father Muller Medical College, Mangalore, Karnataka 575002 India

**Keywords:** Construction worker, Malaria, Knowledge, Mobile populations, Treatment-seeking pattern, Urban

## Abstract

**Background:**

Construction sites are potential breeding places for some species of mosquitoes. Construction workers usually stay at the construction sites, thus being extremely susceptible to malaria. For malaria control, a special focus on them is warranted as they often seek treatment from unregulated, private vendors, increasing their risk of exposure to substandard drugs.

**Objectives:**

To elicit the socio-demographic factors associated with comprehensive malaria knowledge (symptoms, mode of spread, and preventive measures) and treatment-seeking pattern (preferred source and type of treatment) among the construction workers in Mangaluru, India; and, to study the association among their comprehensive malaria knowledge, past suffering from malaria (within 1 year) and treatment-seeking pattern.

**Methods:**

A community based cross-sectional study was conducted in nine randomly selected construction sites of Mangaluru, a high-risk city for malaria with an annual parasite incidence of >2/1000/year, from June–September 2012. A sample size of 132 was estimated assuming at least 30 % of them have satisfactory malaria knowledge, 10 % absolute precision, 95 % confidence level, design effect of 1.5 and 10 % non-responses. A semi-structured interview schedule was used, and knowledge scores were computed. Multivariate linear (for knowledge score) and logistic regressions (for preferred source and type of treatment) were applied.

**Results:**

One hundred and nineteen workers participated in the study (total approached-138). 85 % (n = 101) of them were males. Mean knowledge score was 9.95 ± 3.19 (maximum possible score-16). The majority of them were aware of the symptoms and the mode of malaria transmission. However, <12 % could explain the malaria preventive measures. Females workers (β = −0.281, p = 0.001), self stated malaria within 1 year (β = 0.276, p < 0.001) and who preferred allopathic treatment (β = 0.283, P = 0.001) displayed better knowledge scores. Male workers (AdjOR 7.21, 95 % CI 2.3–22.9) and those with self stated malaria within 1 year (AdjOR 11.21, 95 % CI 2.38–52.8) showed favorable treatment-seeking pattern.

**Conclusions:**

There is an urgent need of intensifying and streamlining of ongoing malaria prevention activities for construction site workers in Mangaluru, India. Emphasizing the gender equity at every stage of programme implementation and addressing their treatment-seeking pattern is essential. Involvement of labour employers and building contractors in this regard is imperative.

## Background

Malaria continues to be a major public health problem in India. Ninety five percent of the country’s population is residing in malaria-endemic areas [1]. The Government of India launched the National Malaria Control Programme in 1953. Within a decade, India had witnessed a tremendous fall in malaria incidence to a mere 50,000 cases per year [2]. Encouraged by this, the strategy was changed to more ambitious National Malaria Eradication Programme in 1958. However, the programme suffered repeated setbacks thereafter due to technical, operational and administrative problems, and cases started increasing again [2]. In response to the scenario, priorities and strategies were changed—i.e. there was a shift from malaria eradication back to malaria control and focus on high-risk areas. Recently, the issue is further complicated by a rise in the incidence and drug resistance of falciparum malaria as well as in the resistance of malaria vector to insecticides [3, 4]. At present, malaria control has been integrated with other vector-borne diseases under National Vector Borne Disease Control Programme (NVBDCP) [5]. India has joined the Asia Pacific Leaders Malaria Alliance (APLMA) to accelerate progress against malaria and to eliminate it in the region by 2030. The APLMA malaria elimination roadmap presents six essential actions to accelerate progress towards malaria elimination. Of the six priorities actions, the first three relate to access to quality medicines to promote elimination and the last three relate to financing [6].

### Urban malaria and construction workers

Since 1970, there has been a surge in urban malaria cases in India. This is largely attributed to unplanned urbanization and large-scale immigration of labourers who have settled in urban slums or areas lacking basic amenities. Immigration is triggered by the phenomenon of rural “push” (for earning livelihood) and an urban “pull” (for availing both healthcare and education) [7]. The Urban Malaria Scheme was launched in 1971 across 131 high-risk Indian cities in a phased manner. Mangaluru (formerly Mangalore) was identified as a high-risk city with an annual parasite incidence (API) of >2 [7]. Employment-driven migration is mainly from the “relatively less developed” states to metropolitan and other large cities, wherein the migrants get absorbed in low-paid jobs in unorganized sectors [8]. According to National Sample Survey Organization of India (2009–2010), 94 % of the employment is in unorganized sector (i.e. 437 million out of 465 million workers). Construction workers constitute nearly 10 % of the unorganized sector [9].

Construction workers are vulnerable to various health hazards. They often settle on lands unfit for settlement and hence are extremely susceptible to malaria and other vector borne diseases. *Anopheles stephensi*, a key malaria vector, prefers to breed in wells, overhead and ground level ater tanks, fountains, masonry tanks and curing water in construction sites [10, 11]. An extremely conducive environment, presence of agent, and the vector, and unprotected construction workers form a perfect epidemiological triad for malaria. In the recent years, Behavior Change Communication (BCC) is being increasingly utilized for informed decision-making and responsive behavior, while enhancing knowledge and awareness about the available malaria control interventions [12]. Information on construction workers’ knowledge and treatment-seeking patterns for malaria, and associated factors is essential for evidence-based design of BCC.

In view of the paucity of studies on construction site workers regarding malaria in Dakshina Kannada district and in the interest of fine-tuning of ongoing BCC activities under NVBDCP, this study was undertaken with the following objectives:

(i) To elicit the socio-demographic factors associated with comprehensive malaria knowledge (symptoms, mode of spread, and preventive measures) and treatment-seeking patterns among construction workers in Mangaluru, India.
(ii) To study the association among comprehensive malaria knowledge, past suffering from malaria (within 1 year) and treatment-seeking pattern among construction workers.

## Methods

### Study setting

This study was conducted in Mangaluru, Dakshina Kannada district, Karnataka state, India from June–September 2012. Mangaluru is a picturesque city located at 12.87°N 74.88°E. It is situated on the shore of Arabian Sea and is also the district headquarters with a population of 0.5 million. It has a tropical monsoon climate and the average annual rainfall is about 4000 mm. It is considered as a malaria high-risk city and the district is classified as highly endemic with an API of 2–5 [7, 13]. Mangaluru is one of the only three districts (out of 34) in Karnataka with an API of >2. This level of endemicity is largely attributed to its geographical tenure, heavy rainfall, construction activities and poor environmental conditions. Owing to the enormous spurt in construction activities and rapid industrialization there has been a lot of labour immigration to Mangaluru from various parts of the state and India [14]. An active surveillance activity of construction workers (n = 1310 smears) in Mangaluru (2004–2005), revealed a malaria slide positivity rate of 25.3 % [15]. The role of *An. stephensi* in malaria transmission is reported in Mangaluru [10, 11]. Five municipal corporation and two government hospitals, as well as three local medical colleges cater to the health needs of urban population.

### Study design and sample

A community based cross-sectional study was conducted in Mangaluru, India from June–September 2012. A sample size of 120 was estimated using Epi Info™ 7.1.5 software based on the following: expected total study population of 4800 in Mangaluru city, assuming at least 30 % of the construction site workers have satisfactory knowledge of malaria, 10 % absolute precision, 95 % confidence level, a design effect of 1.5 due to multistage random sampling [16]. The sample size was inflated to 132 expecting 10 % non-responses.

Mangaluru city is divided into three zones by Mangaluru Urban Development Authority, a regulatory body pertaining to construction of commercial buildings. Keeping the resource constrains in mind, three multi-storey construction sites from each zone were selected by simple random sampling. Inclusion criteria were: age ≥18 years, has been working as a construction worker for a minimum period of 6 months and consenting for voluntary participation. One of the authors went to the selected nine sites and enlisted 286 (38 female and 248 male) eligible construction workers. The required number of sample was selected by stratified random sampling with proportional allocation, keeping the sex ratio in mind.

### Study tool

A semi structured interview schedule was prepared to elicit the socio-demographic profile, self stated history of malaria in the last 1 year, resident at the construction site, knowledge of malaria spread and clinical features, awareness about mosquito breeding places, preventive measures and availability of free diagnosis and treatment in government hospital/healthcare setup. Treatment-seeking patterns were also elicited by noting ‘top-of-the-mind’ responses (instantaneous response to an unprompted question) for the following: what was the preferred source and type of treatment, if he/she had suffered from malaria? Or what would be the preferred source and type treatment if he/she suffers from malaria. And the responses were categorised as government/public or private healthcare facility and allopathic, ayurveda, homeopathy or siddha for the preferred source and the type of treatment, respectively.

A scoring system was developed to assess the respondents’ comprehensive malaria knowledge. One point was awarded for every correct response and none for every wrong response. The maximum possible score was 16. To score maximum, respondents should have heard the term ‘malaria’ (one point), enumerate the common symptoms i.e. fever (one point), chills and rigors (one point), sweating (one point), head/body ache and vomiting (one point); know that malaria is transmitted by mosquitoes (one point); aware of common mosquito breeding places i.e. water logged area/fountain/natural water reservoir (one point), uncovered water tank/storage(one point); know about preventive measures i.e. use of bed nets (one point), avoid water logging and keep surroundings clean or proper covering of stored water or filling of the breeding places (one point), indoor spray (one point), screening of the houses with wire mesh (one point), wearing clothes that cover maximum surface area of the body (one point), use of mosquito repellents i.e. ointment/coils/liquidators (one point), use of guppy/gambusia larvicidal fish in ornamental tanks, fountains etc. (one point); aware of free diagnosis and treatment in government healthcare facility (one point).

Validation of the study tool was done by three research experts. Before the data collection, pretesting of the questionnaire and the scoring system was done on 10 construction workers, in a randomly selected construction site, to ensure that questions were easily understood. Cronbach’s alpha of 0.768 endorsed the internal consistency of the scoring system [17]. The pre-tested construction site was dropped while randomly selecting nine sites. All the interviews were conducted by the second author to avoid inter-observer bias.

### Statistical analysis

Data was analysed using Statistical Package for the Social Sciences (SPSS) for Windows, Version 16.0. Chicago, SPSS Inc. Results were expressed as frequencies and proportions for categorical variables and mean and standard deviations for continuous variables. Normality test of knowledge score displayed near normal distribution. Chi square, Fisher’s exact and unpaired t tests were applied to assess the significant differences across study variables. Two sided p < 0.05 was considered as statistically significant. Multivariate linear (for knowledge score) and logistic regressions (for other categorical variables) were run for significant associations by univariate analysis (p < 0.05), to examine the simultaneous impact of several factors on knowledge score and treatment-seeking pattern. Workers mean knowledge score, regression coefficients for knowledge scores and adjusted odds ratios (AdjOR) for treatment-seeking pattern across various independent variables were the key outcome measures.

### Ethical approval

The Ethics committee of Yenepoya University, Mangaluru, India approved the study protocol (YUEC132/06/07/2013) and necessary permission was taken from Mangaluru Urban Development Authority. The study protocol followed the tenets of the Declaration of Helsinki. Informed written consent was taken from all the respondents for voluntary participation either in *kannada* (local language) or in *hindi* (national language) for indigenous and migrant workers, respectively.

## Results

A total of 138 eligible construction workers were approached in nine randomly selected construction sites and 119 participated in the study (response rate 86.2 %). Out of 119 workers, 18 (15 %) were females and 101 (85 %) were males and male:female ratio was 6.6. Nearly half (n = 52, 43.7 %) of them were in the age group of 21–30 years and their mean age was 28.41 ± 11.37 years (Table 1). Most (n = 116, 97.5 %) of them were migrant workers. Workers from scheduled caste (SC) category were the majority (n = 58, 48.7 %) followed by other backward class (OBC) category (n = 31, 26.1 %). Among the respondents, 49 (41.2 %) were illiterates and illiteracy was significantly (p = 0.004) higher among females compared to males (72.2 % vs. 35.6 %). Almost three fourths of them (n = 89, 74.8 %) were residing at the construction site. Except one, all the female workers (n = 17, 94.4 %) were married and were working together with their husbands at the construction sites. Nearly half of the workers (n = 55, 46.2 %, 95 % CI 37.2–55.2 %) reported that they suffered from malaria within a duration of 1 year and it was significantly high (p = 0.019) among those who were staying at the construction site (52.8 vs. 26.7 %). Proportion wise differences in self stated past suffering from malaria within 1 year across other studied socio-demographic variables were statistically not significant (p > 0.05) (Table 2).

**Table 1:** Socio-demographic profile and self stated history of malaria within 1 year among the construction workers in Mangaluru, India, June–September 2012 (n = 119)

Study variable	Male		Female		Total	
	n	%	n	%	n	%
Age group (years)						
18–20	26	25.7	6	33.3	32	26.9
21–30	46	45.5	6	33.3	52	43.7
31–40	16	15.8	2	11.1	18	15.1
41–50	8	7.9	2	11.1	10	8.4
>50	5	5.0	2	11.1	7	5.9
Type of worker						
Local	3	3.0	0	0	3	2.5
Migrant	98	97.0	18	100	116	97.5
Caste category						
Scheduled caste	43	42.6	15	83.3	58	48.7
Scheduled tribe	7	6.9	0	0	7	5.9
Other backward class	29	28.7	2	11	31	26.1
General caste	22	21.8	1	5.6	23	19.3
Literacy status						
Illiterate	36	35.6	13	72.2	49	41.2
Up to primary	29	28.7	3	16.7	32	26.9
Up to high school	26	25.7	2	11.1	28	23.5
Above high school	10	9.9	0	0	10	8.4
Marital status						
Married	52	51.5	17	94.4	69	58
Unmarried	49	48.5	1	5.6	50	42
Stay at construction site						
Yes	75	74.3	14	77.8	89	74.8
No	26	25.7	4	22.2	30	25.2
History of malaria within 1 year						
Yes	49	48.5	6	33.3	55	46.2
No	52	51.5	12	66.7	64	53.8
Total	101	100	18	100	119	100

**Table 2:** Association between self stated history of malaria within 1 year and socio-demographic variables of the construction workers in Mangaluru, India, June–September 2012 (n = 119)

Study variable	History of malaria within 1 year				p
	No		Yes		
	n	%	n	%	
Age (years)					
<25	31	53.4	27	46.6	1.000
≥25	33	54.1	28	45.9	
Gender					
Female	12	66.7	6	33.3	0.234
Male	52	51.5	49	48.5	
Type of worker					
Local	1	33.3	2	66.7	0.595^b^
Migrant	63	54.3	53	45.7	
Marital status					
Unmarried	22	44	28	56	0.608
Married	42	60.9	27	39.1	
Literacy status					
Illiterate	37	52.8	33	47.2	0.809
Literate	27	55.1	22	44.9	
Caste category^c^					
SC/ST	38	58.5	27	41.5	0.261
General/OBC	26	48.2	28	51.8	
Stay at construction site					
No	22	73.3	8	26.7	0.013^a^
Yes	42	47.2	47	52.8	

^a^Significant (p < 0.05)

^b^Fisher’s Exact Test

^c^ *SC* scheduled caste *ST* scheduled tribe, *OBC* other backward caste; df = 1 for all variables

A government/public healthcare facility was the preferred source for malaria treatment among them (n = 83, 69.7 %). Most of those who preferred the private sector were unaware of the availability of free diagnosis and treatment in government healthcare facility. Many of them (n = 98, 82.4 %) favoured allopathic/modern medicine over complementary systems.

Respondents’ mean knowledge score was 9.95 ± 3.19 (range 5–13). Substantial correct responses were recorded for ‘heard of malaria’ (n = 95, 80 %); clinical symptoms i.e. fever (n = 84, 70.6 %), chills and rigors (n = 67, 56.3 %), body/headache and vomiting (n = 58, 48.7 %); mosquitoes transmit malaria (n = 72, 60.5 %); mosquitoes breed in water logged area (n = 61, 51.3 %) and free diagnosis and treatment in government hospital (56.3 %). Over 1/4th (26.1 %) told about the use of mosquito coils, however, only few of them (< 12 %) could tell about other preventive measures i.e. use of bed nets, screening of the houses with wire mesh, wearing clothes covering maximum body surface area.

The mean malaria knowledge scores were significantly high (p < 0.05) among male workers, literates, those who suffered from malaria within 1 year, preferred government healthcare facility and modern medicine/allopathic treatment when compared to their counterparts (Table 3). Differences in mean knowledge scores among other socio-demographic factors like age, marital status, caste category, stay at the construction site and migration were statistically not significant (p > 0.05).

**Table 3:** Construction workers’ mean malaria knowledge scores according to their socio-demographic variables and treatment-seeking pattern in Mangaluru, India, June–September 2012 (n = 119, maximum possible score = 16)

Study variable	n	Mean knowledge score	SD	p
Age (years)				
<25	58	10.0	3.31	0.772
≥25	61	9.9	3.09	
Gender				
Female	18	6.7	2.77	<0.001^a^
Male	101	10.5	2.91	
Type of worker				
Migrant	116	9.9	3.20	0.281^b^
Local	3	12.0	2.65	
Stay at construction site				
Yes	89	10.1	3.03	0.533
No	30	9.6	3.68	
History of malaria within 1 year				
Yes	55	11.3	2.71	<0.001^a^
No	64	8.8	3.11	
Literacy status				
Illiterate	49	8.9	3.19	0.002^a^
Literate	70	10.7	3.00	
Caste category^c^				
SC/ST	65	9.6	3.67	0.139
General/OBC	54	10.4	2.46	
Marital status				
Unmarried	50	10.5	2.92	0.097
Married	69	9.5	3.34	
Preferred source of treatment				
Government	83	10.6	2.9	0.004^a^
Private	36	8.6	3.5	
Preferred treatment				
Allopathic	98	10.7	2.9	<0.001^a^
Others	21	6.5	1.9	

^a^Significant (p < 0.05)

^b^Mann–Whitney U test

^c^ *SC* scheduled caste, *ST* scheduled tribe, *OBC* other backward caste

Significantly (p < 0.05) higher number of males (87.1 and 77.2 %) and literates (90 and 78.6 %) preferred allopathic treatment and public health sector facility for malaria, respectively. Similarly, a higher number of those who had suffered from malaria within 1 year(96.4 %) preferred allopathic treatment (p < 0.001) (Table 4).

**Table 4:** Construction workers’ preferred source and type of treatment according their socio-demographic profile and self stated history of malaria within 1 year in Mangaluru, India, June–September 2012 (n = 119)

Study variable	Preferred treatment source					Preferred treatment type				
	Public sector		Private sector		p	Allopathic		Others		p
	n	%	n	%		n	%	n	%	
Age (years)										
<25	42	72.4	16	27.6	0.537	50	86.2	8	13.8	0.282
≥25	41	67.2	20	32.8		48	78.7	13	21.3	
Gender										
Female	5	27.8	13	72.2	<0.001^a^	10	55.6	8	44.4	0.004^a, b^
Male	78	77.2	23	22.8		88	87.1	13	12.9	
Type of worker										
Local	1	33.3	2	66.7	0.217^b^	3	100.0	0	0.0	1.00^b^
Migrant	82	70.7	34	29.3		95	81.9	21	18.1	
Marital status										
Unmarried	38	76.0	12	24.0	0.206	44	88.0	6	12.0	0.167
Married	45	65.2	24	34.8		54	78.3	15	21.7	
Literacy status										
Illiterate	28	57.1	21	42.9	0.012^a^	35	71.4	14	28.6	0.009^a^
Literate	55	78.6	15	21.4		63	90.0	7	10.0	
Caste category^c^										
SC/ST	45	69.2	20	30.8	0.893	51	78.5	14	21.5	0.222
General/OBC	38	70.4	16	29.6		47	87.0	7	13.0	
History of malaria within 1 year										
Yes	40	72.7	15	27.3	0.512	53	96.4	2	3.6	<0.001^a^
No	43	67.2	21	32.8		45	70.3	19	29.7	

^a^Significant (p < 0.05)

^b^Fisher’s Exact Test

^c^ *SC* scheduled caste, *ST* scheduled tribe, *OBC* other backward caste; df = 1 for all variables

Multiple linear regression analysis (Table 5) showed that female workers (β = −0.281, p = 0.001), those who those who reported suffering from malaria within 1 year (β = 0.276, p < 0.001) and those who preferred allopathic/modern medicine (β = 0.283, p = 0.001) displayed higher knowledge scores. The applied linear regression model could explain a variance of 41.7 % in the mean knowledge score (F = 16.18, p < 0.01) which was not high but within acceptable limits. Similarly, multiple logistic regression analysis revealed that history of malaria within 1 year (AdjOR 11.21, 95 % CI 2.38–52.84) and male worker (AdjOR 7.21, 95 % CI 2.26–22.99) were the predictors of preference to allopathic treatment and public healthcare facility, respectively (Table 6).

**Table 5:** Multiple linear regression analysis for association between malaria knowledge scores and socio-demographic parameters and treatment-seeking patterns of construction workers in Mangaluru, India, June–September 2012 (n = 119)

Predictor variable	B	SE	β	t	p	95 % CI	
Female worker	−2.492	0.712	−0.281	−3.498	0.001^a^	−3.903	−1.081
Illiteracy	−0.836	0.492	−0.129	−1.700	0.092	−1.811	0.138
History of malaria within 1 year	1.759	0.491	0.276	3.585	<0.001^a^	0.787	2.732
Prefer allopathic treatment	2.365	0.717	0.283	3.298	0.001^a^	0.944	3.786
Prefer public healthcare facility	0.114	0.578	0.016	0.197	0.844	−1.032	1.260
Constant	7.830	–	–	–	–	–	–

*SE* standard error; Coefficient of correlation (r = 0.646); coefficient of determination (r^2^ = 0.417)

Dependent variable ‘malaria knowledge score’ (range 0–16)

^a^Significant (p < 0.05)

**Table 6:** Crude and adjusted odds ratios (OR) for the association between socio-demographic parameters and treatment-seeking patterns among construction workers in Mangaluru, India, June–September 2012 (n = 119)

	Prefer public healthcare facility				Prefer allopathic treatment			
	Crude OR	95 % CI	Adjusted^a^ OR	95 % CI	Crude OR	95 % CI	Adjusted^a^ OR	95 % CI
Age (years)								
<25	1.280	0.584–2.809	–	–	1.692	0.644–4.45	–	–
≥25	1				1			
Gender								
Male	8.817	2.844–27.33^b^	7.207	2.259–22.98^b^	5.42	1.81–16.2	3.496	0.95–12.9
Female	1		1		1		1	
Type of worker								
Migrant	4.823	0.423–54.985	–	–	0.634	0.032–12.7	–	–
Local	1				1			
Marital status								
Married	0.592	0.262–1.339	–	–	0.490	0.176–1.37	–	–
Unmarried	1				1			
Literacy status								
Literate	2.75	1.231–6.143^b^	1.984	0.831–4.736	3.6	1.33–9.76	2.946	0.945–9.188
Illiterate	1		1		1		1	
Caste category^c^								
SC/ST	0.947	0.431–2.08	–	–	0.542	0.20–1.46	–	–
General/OBC	1				1			
History of malaria within 1 year								
Yes	1.302	0.591–2.87	–	–	11.19	2.47–50.66	11.21	2.378–52.84^b^
No	1				1		1	

Nagelkerke R Square = 0.21(Prefer public healthcare facility)

Nagelkerke R Square = 0.33 (Prefer allopathic treatment)

^a^By binomial logistic regression

^b^Significant (p < 0.05)

^c^ *SC* scheduled caste, *ST* scheduled tribe, *OBC* other backward caste

## Discussion

### Main findings

Knowledge of the cause, symptoms and availability of free diagnostic and treatment facility in public health sector for malaria was satisfactory in the study population. However, the knowledge of simple malaria preventive measures was unsatisfactory. Male worker and those who reported suffering from malaria within 1 year were associated with better comprehensive malaria knowledge and favorable treatment-seeking pattern.

In urban India, approximately 86/1000 employed people are construction workers and constitute 10 % of the workforce in the unorganized sector [18]. Despite the high risk of malaria and substantial number, construction workers are often overlooked. This paper attempts to explore this knowledge gap by using data from a target population-based cross-sectional study.

The present study also affirms the workers’ high vulnerability to malaria (46.2 % had past history of malaria). The risk is further augmented by unsatisfactory levels of comprehensive malaria knowledge and, hence, potentially poor utilization of protective measures. Poor knowledge could largely be attributed to limited levels of health education activities for them. This study and others [19–22] suggest that, a substantial proportion of people living in endemic areas are familiar with the term “malaria”, its symptoms and mode of spread. Hence, variations in comprehensive malaria knowledge are observed in awareness of mosquito breeding places and feasible preventive measures and their appropriate implementation. Diverse factors, such as age, gender, education, economic status, personal experience of malaria, transmission level, treatment availability and accessibility, etc. have been mentioned to explain such variations in many ‘knowledge, attitude and practice’ studies [19–23].

Very poor knowledge of preventive measures detected in this study, especially bed net use, makes immediate attention and action necessary. Similar observation is reported in studies from New Delhi, India [23], Haiti [24], Turkey [25], Ethiopia [26] and Iran [27]. However, higher bed net knowledge was reported in Bangladesh [28], Nepal [29] and Ghana [30]. *Anopheles stephensi* rests mainly indoors and has nocturnal peak biting activity. Hence, in urban settings personal protection measure with insecticide-treated nets (ITNs), especially with synthetic pyrethroids is a key strategy for malaria control [12]. In addition, the cost effectiveness of insecticide treated bed nets has been widely reported [31–33]. NVBDCP also endorses rapid scale up of insecticide treated bed net coverage (minimum 80 %) through mass distribution campaign in high risk areas and free re-treatment of plain nets with synthetic pyrethroid [12]. However, such actions must be backed up by intensified BCC activities to enhance the community acceptability and sustainable use [34].

In most part of the world, women are the primary caretakers of the family members and provide majority of treatment to sick family members [35]. Hence, women’s awareness could go a long way in providing timely treatment and implementation of the available malaria prevention tools at household level. Poor knowledge and unfavourable treatment-seeking patterns among female workers in this study could be attributed to higher level of illiteracy (72.2 %) and lack of access to information. Considering the patriarchal norms of the Indian society, this is not a surprise finding. Illiteracy may affect their ability to recognize the symptoms of malaria and knowledge of available treatment [35]. A study in Nigeria reported that higher levels of education were associated with better knowledge and malaria treatment and preventive practice among women [36]. Women disfavouring knowledge level and health-seeking behaviours are reported in Bangladesh [28, 37]. Although, women are the primary caretakers in the family but, decisions about seeking are made by men. Available evidence also suggests that even women focused interventions may not increase the access of quality healthcare for women, if the gender issues are not explicitly addressed by the programme [38, 39]. Therefore, gender equity focus at every stage of programme implementation and emphasis on informal communication methods (e.g., folk songs, people’s theatre, street play) is needed [28].

For malaria control, a special focus on migrants and mobile populations is warranted as these groups often seek treatment from unregulated, private vendors, increasing their risk of exposure to substandard drugs [40]. However, workers’ preference to allopathy (82.4 %) and public health sector (69.7 %) for the treatment in our study is admirable and is to be endorsed. Preference for non-allopathic treatment was a predictor of poor knowledge in this study. The availability of free diagnosis and curative treatment in public health sector should be canvassed for further improvement. Contrary to this, many studies from African countries reported non-public sources as the main sources of treatment [41–44]. Another study from Kenya [45] reported that public sector providers’ better knowledge and practices on treatment and dosing regimen with policy-recommended anti-malarials than private and not-for-profit sectors.

The effect of “opportunistic health education” at a healthcare facility was evident in this study as the history of malaria within 1 year was a predictor of better knowledge. This is corroborated by the findings of Tyagi et al. [23]. More efforts are required at primary prevention level so that adequate preventive knowledge is imparted to avoid the onset of malaria.

Many initiatives have been undertaken by the Mangaluru Municipal Corporation and the district health authority to curb malaria. Active surveillance of construction site workers, random inspection of construction sites, guidelines for source reduction and protection of workers at construction sites, edifying a legal framework for employers and building contractors, are the key strategies by Mangaluru Municipal Corporation [46, 47]. However, the extent of target population coverage, sustainability, involvement of the labour employers and building contractors and acceptability by the construction workers, are the debatable issues that need to be addressed in order to foresee the impact.

The Strategic Plan for Malaria Control in India (2012–2017) targets an API of <1 by the end of 2017 [12]. Mass screening of migrant labourers, indoor spray and insecticide-treated bed nets are the key strategies for high-risk areas like construction or development project sites. Insufficient use of educational material, especially in local languages, and inadequate efforts toward community mobilization are pointed out in the SWOT (strengths, weaknesses, opportunities and threats) analysis of NVBDCP [11]. In this regard, the findings of the present study are pertinent. It emphasizes on intensifying and fine tuning of malaria BCC strategy for construction workers. Better knowledge and positive attitudes influence health-seeking behavior and enhance the effectiveness of the control measures [48, 49].

## Limitations

Owing to the cross sectional study design, associations observed between the study variables may not be causal. We relied on construction worker’s recall for the history of malaria within 1 year and could not verify the diagnosis (none of them had any records). This may have resulted in recall bias. Considering the relatively small sample size (due to lack of resources) and workers’ frequent mobility, the studied sample may not represent the construction workers’ population in Mangaluru.

## Conclusions

The knowledge of malaria preventive measures among the construction workers in Mangaluru was unsatisfactory, although a substantial proportion of them were aware of the cause, symptoms and free diagnosis and treatment in public health sector for malaria. There is an urgent need of intensifying and streamlining of ongoing malaria BCC activities for construction site workers in Mangaluru, India. Emphasizing the gender equity at every stage of programme implementation and addressing their treatment-seeking pattern is required. Sufficient use of the health educational material in local languages and effective use informal communication methods for health education are needed. Involvement of labour employers and building contractors in this regard is imperative.

## Abbreviations

API: annual parasite incidence
BCC: behavior change communication
CI: confidence interval
NVBDCP: National Vector Borne Disease Control Programme
SPSS: Statistical Package for Social Sciences
SWOT: strength weakness opportunity and threat
