# Erratum to: Construction site workers’ malaria knowledge and treatment-seeking pattern in a highly endemic urban area of India

**DOI:** 10.1186/s12936-016-1472-6

**Published:** 2016-08-16

**Authors:** Siddharudha Shivalli, Sudarshan Pai, Kibballi Madhukeshwar Akshaya, Neevan D’Souza

**Affiliations:** 1 Department of Community Medicine, Yenepoya Medical College, Yenepoya University, Mangalore, Karnataka 575018 India; 2 Department of Community Medicine, Father Muller Medical College, Mangalore, Karnataka 575002 India

## Erratum to: Malar J (2016) 15:168 DOI 10.1186/s12936-016-1229-2

After publication of the original article [1], the authors noted an error in the reporting of findings in the Results sections of both the abstract and the article.

The association between gender and malaria knowledge score was wrongly reported. The correct interpretation of multiple linear regression analysis is that female workers (β = −0.281, p = 0.001) displayed poor malaria knowledge scores when compared to males.

The Results section should therefore have read as follows:

Multiple linear regression analysis (Table 5) showed that female construction-site workers (β = −0.281, p = 0.001) displayed poor malaria knowledge scores when compared to males. Workers who reported suffering from malaria within 1 year (β = 0.276, p < 0.001) and those who preferred allopathic/modern medicine (β = 0.283, p = 0.001) displayed higher knowledge scores.
